# A comparison between the 2010 and 2005 basic life support guidelines during simulated hypogravity and microgravity

**DOI:** 10.1186/2046-7648-2-11

**Published:** 2013-04-01

**Authors:** Thais Russomano, Justin H Baers, Rochelle Velho, Ricardo B Cardoso, Alexandra Ashcroft, Lucas Rehnberg, Rodrigo D Gehrke, Mariana K P Dias, Rafael R Baptista

**Affiliations:** 1 Microgravity Centre, School of Engineering, PUCRS, Porto Alegre 90619-900, Brazil; 2 Centre of Human and Aerospace Physiological Sciences, School of Biomedical Sciences, KCL, London WC2R 2LS, UK

**Keywords:** Basic life support, CPR guidelines, Hypogravity, Microgravity

## Abstract

**Background:**

Current 2010 terrestrial (1G_z_) CPR guidelines have been advocated by space agencies for hypogravity and microgravity environments, but may not be feasible. The aims of this study were to (1) evaluate rescuer performance over 1.5 min of external chest compressions (ECCs) during simulated Martian hypogravity (0.38G_z_) and microgravity (μG) in relation to 1G_z_ and rest baseline and (2) compare the physiological costs of conducting ECCs in accordance with the 2010 and 2005 CPR guidelines.

**Methods:**

Thirty healthy male volunteers, ranging from 17 to 30 years, performed four sets of 30 ECCs for 1.5 min using the 2010 and 2005 ECC guidelines during 1G_z_, 0.38G_z_ and μG simulations (Evetts-Russomano (ER) method), achieved by the use of a body suspension device. ECC depth and rate, range of elbow flexion, post-ECC heart rate (HR), minute ventilation (*V*_E_), peak oxygen consumption (VO_2_peak) and rate of perceived exertion (RPE) were measured.

**Results:**

All volunteers completed the study. Mean ECC rate was achieved for all gravitational conditions, but true depth during simulated microgravity was not sufficient for the 2005 (28.5 ± 7.0 mm) and 2010 (32.9 ± 8.7 mm) guidelines, even with a mean range of elbow flexion of 15°. HR, *V*_E_ and VO_2_peak increased to an average of 136 ± 22 bpm, 37.5 ± 10.3 L·min^−1^, 20.5 ± 7.6 mL·kg^−1^·min^−1^ for 0.38G_z_ and 161 ± 19 bpm, 58.1 ± 15.0 L·min^−1^, 24.1 ± 5.6 mL·kg^−1^·min^−1^ for μG from a baseline of 84 ± 15 bpm, 11.4 ± 5.9 L·min^−1^, 3.2 ± 1.1 mL·kg^−1^·min^-1^, respectively. RPE was the only variable to increase with the 2010 guidelines.

**Conclusion:**

No additional physiological cost using the 2010 basic life support (BLS) guidelines was needed for healthy males performing ECCs for 1.5 min, independent of gravitational environment. This cost, however, increased for each condition tested when the two guidelines were compared. Effective ECCs were not achievable for both guidelines in simulated μG using the ER BLS method. This suggests that future implementation of an ER BLS in a simulated μG instruction programme as well as upper arm strength training is required to perform effective BLS in space.

## Background

Human exploration of space is curtailed by the physiological and technical impact of reduced gravity. Nevertheless, it has provoked a fascination in mankind as limitless as the void of space itself. Aerospace medicine and physiology are evolving in tandem with explorer-class missions to accommodate the challenges associated with maintaining the safety, health and optimum performance of astronauts during spaceflights.

All organ systems are affected by exposure to extra-terrestrial environments. Alterations to cardiovascular physiology with reduced gravity manifest acutely and chronically [1]. Reduced-gravity environments cause the cardiovascular system to undergo adaptive functional and structural changes. Microgravity induces a reduction in hydrostatic pressure, causing a cephalic redistribution of blood and body fluids. This headward shift is responsible for the ‘puffy-face & bird-leg’ appearance of astronauts in space. The cardiovascular system adapts to microgravity by reducing blood volume by approximately 20%, which is in part responsible for the orthostatic intolerance commonly found post-spaceflight. A reduction in heart size was also observed in microgravity [2]. However, based on data from space missions, it is suggested that such cardiovascular alterations do not lead to important cardiac dysfunction or dysrrhythmias. Therefore, the possibility of cardiac deconditioning developing into a life-threatening condition, such as a cardiac arrest, during short to moderate spaceflights is approximately 1% per year [3]. Nevertheless, with space agencies shifting their emphasis to lunar return missions and the eventual human exploration of Mars, the likelihood for cardiovascular issues to manifest themselves will be further enhanced with increasing space mission length.

An explorer-class mission to Mars will require approximately 2.4 years for completion: a 6-month flight to Mars, an approximate 500-day surface stay, and a 6-month return flight to Earth [4]. The cumulative and interactive effects of physiological problems from a long-term spaceflight could be potentially devastating for crewmembers. Prolonged exposure to reduced gravity may result in altered heart conduction and repolarisation, predisposing astronauts to cardiac dysrrhythmias [5]; electrical heart instability, in conjunction with encountered biodynamic stressors, presents the disturbing possibility of cardiac arrest in astronauts partaking in lengthy missions.

Further to exploration-class missions, the global private sector is having a greater influence on space ventures. The introduction of civilian tourist space travel broadens the population who may be subjected to the pertinent aspects of cardiovascular risks associated with spaceflight. Survey data show the demographics expected for suborbital spaceflight participants to be 70% male with an average of 57 years of age, 22% of which were older than 65 years [6]. This suggests that the expected population engaging in civilian spaceflight will be more likely to harbour subclinical cardiovascular conditions, hence increasing the probability of a cardiac event. Currently, international space institutions are refraining from imposing safety regulations, stating that there are no medical requirements for space tourism passengers and that only minimum training is required on how to respond to emergency situations [7].

Effective management of acute and chronic medical emergencies, such as basic life support (BLS), is vital on missions to ensure astronaut and tourist safety. External chest compressions (ECCs) constitute the core of BLS and must continue until advanced life support (ALS) can commence to maintain adequate perfusion to vital organs. The collaborative algorithm between the American Heart Association and the European Resuscitation Council for adult BLS delineates key steps required for effective terrestrial cardiopulmonary resuscitation (CPR) and was updated in 2010 [8]. These new guidelines place more emphasis on ECCs than ventilation. The previous airways-breathing-circulation ‘A-B-C’ algorithm has been altered to ‘C-A-B’. This ensures rapid blood distribution to target areas whilst oxygen saturation is sufficiently high. It is now essential to perform ECCs of adequate depth (minimum 50 mm) and rate (100 compressions·min^−1^) [8].

Terrestrial (1G_z_) CPR guidelines have been advocated by international space agencies for hypogravity and microgravity environments. Nonetheless, performing ECCs during spaceflight is more challenging due to reduced gravity [9]. Previous studies have shown the 2005 CPR guidelines to be feasible for simulated hypogravity and microgravity conditions. However, current guidelines, which require deeper ECCs, may not be feasible without compromising the rescuer's health and may go beyond the rescuer's physical capability; therefore, a comparison between the 2005 and 2010 CPR guidelines in hypogravity and microgravity environments is needed.

This investigation aimed to evaluate rescuer performance over 1.5 min of ECCs during simulated Martian hypogravity and microgravity in relation to 1G_z_ and additionally compare the physiological costs of conducting ECCs in accordance with the 2005 and 2010 CPR guidelines. It was hypothesised that current ECC depth and frequency guidelines should be achievable for all simulated gravitational conditions. However, the 2010 ECC guidelines were expected to be more physiologically demanding in proportion to the reduction in simulated gravity.

## Methods

### Study design

The protocol included performing four sets of 30 ECCs over a period of 1.5 min in accordance to the 2005 and 2010 CPR guidelines during 1Gz, ground-based Martian hypogravity (0.38G_z_) and microgravity (μG) simulations at the John Ernsting Aerospace Physiology Laboratory, Microgravity Centre, Pontifícia Universidade Catolica do Rio Grande do Sul (PUCRS), Brazil. The study employed a within-volunteer repeated measures design, with each volunteer being their own control. The order of simulated gravitational conditions and CPR guidelines were randomised. The study protocol was approved by the Ethics and Research Committees of PUCRS.

### Volunteers

A total of 30 healthy male volunteers, ranging from 17 to 30 years of age, served as rescuers performing CPR. They were recruited on a voluntary basis and signed a consent form prior to the beginning of the study.

### Equipment and materials

A standard CPR mannequin (Resusci Anne Skill Reporter, Laerdal Medical Ltd., Orpington, UK) was modified to include a linear displacement transducer capable of measuring ECC depth and rate. The mannequin's chest steel spring depressed 1 mm with every 1 kg of weight that was applied to it. Real-time feedback of each ECC was provided to the volunteers via a modified electronic guiding system with a light-emitting diode (LED) display. The LED display consisted of a series of coloured lights that indicated depth of ECCs (red, 0–39 mm; yellow, 40–49 mm; green, 50–60 mm). An ECC rate of 100 compressions·min^−1^ was established using an electronic metronome. A 6-s interval between each ECC set represented the time taken for two mouth-to-mouth ventilations.

**Figure 1:**
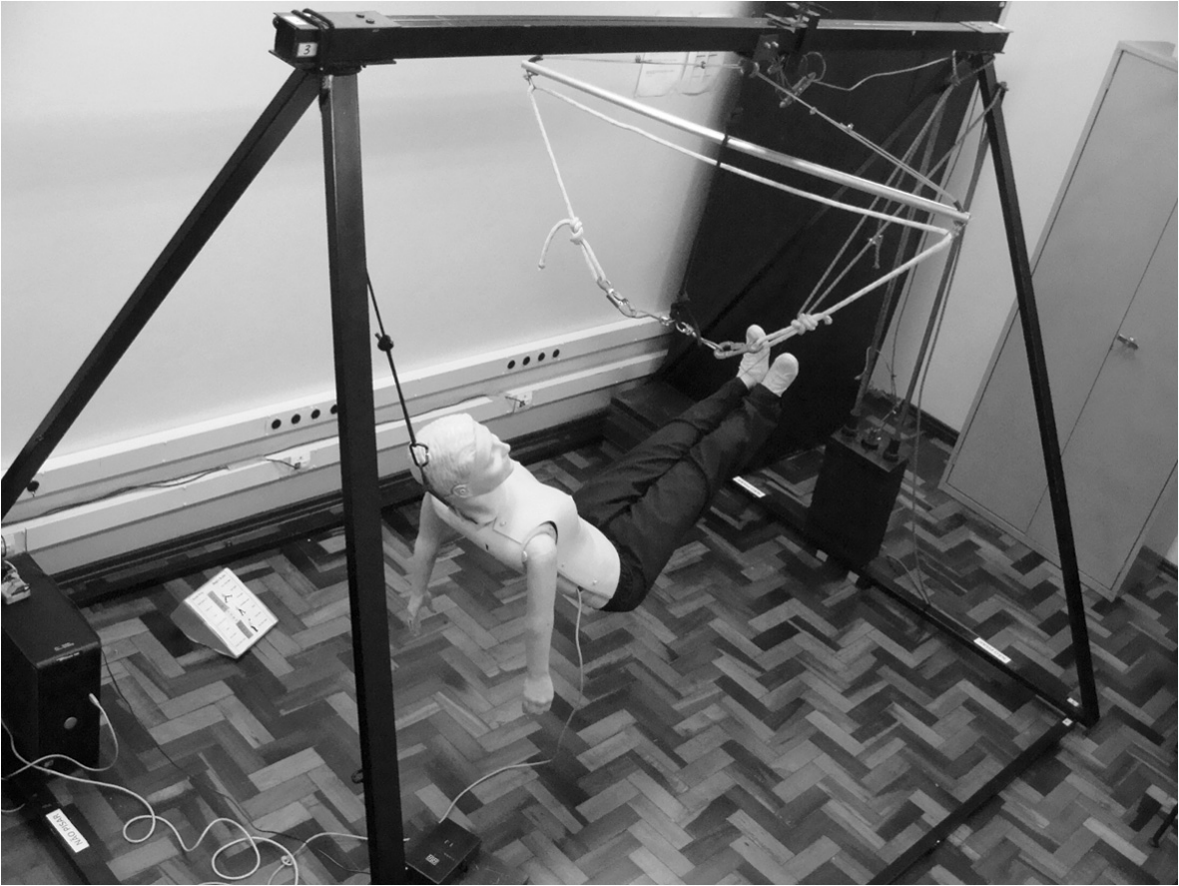
Body suspension device with mannequin fully suspended simulated microgravity.

A custom-built body suspension device (BSD) was used to simulate reduced gravitational fields (developed by the Microgravity Centre, PUCRS). It is pyramidal in shape and consists of carbon steel bars of 6 cm × 3 cm thickness (base area, 300 cm × 226 cm; height, 200 cm). It comprises of a body harness and counterweight system made of 20 bars of 5 kg each (Figure 1).

For simulated 0.38G_z_, the steel cable connected the counterweights through a pulley system to the harness worn by the volunteer. The necessary counterweights were calculated using Equations 1 and 2 [10]:

(1) $$\mathrm{RM}=\frac{0.6\mathrm{BM}\times \mathrm{SGF}}{1G}$$

(2) CW=0.6BM−RM

where RM is the relative mass (in kg), 0.6BM is the percentage of total body mass, SGF is the simulated gravitational force (m·s^−2^), 1G = 9.81 m·s^−2^ and CW is the counterweight (in kg).

During the performance of ECCs, the mannequin was placed supine on the floor with the volunteer adopting the terrestrial CPR position.

For simulated μG, volunteers were suspended by the body harness via the use of the steel cross bar (1205.0 mm × 27.5 mm). A static nylon rope was attached to the steel wiring of the cross bar, with carabineers fastened at each end. These were clipped to corresponding hip attachments of the body harness. A safety carabineer was also attached to the volunteer's back.

The mannequin was fully suspended to allow the performance of the Evetts-Russomano (ER) BLS technique. In order to perform the ER technique, the volunteer places his left leg over the mannequin's right shoulder and his right leg around the torso and across the back of the mannequin. The left and right ankles cross in the inter-scapula area of the mannequin for added stability. The application of force to the chest of the mannequin will then be countered by the volunteer's legs and feet and is achieved by the flexion and extension of the volunteer's arms [11].

Angle of elbow flexion was measured using a custom-built electrogoniometer on the volunteer's dominant arm (developed by the Microgravity Centre, PUCRS). The electrogoniometer consisted of two aluminium bars (200.0 mm × 20.0 mm × 3.0 mm) covered with rubber material and was fastened over the volunteer's lateral epicondyle via a series of straps; this allowed the change in flexion/extension (from 0° to 90°) to be accurately measured. The device was connected with a linear 10 kΩ potentiometer and powered by a 5-V power source.

An Aerosport VO2000 analyser (MedGraphics, Saint Paul, MN, USA) recorded minute ventilation (*V*_E_) and oxygen consumption per minute (VO_2_). VO_2_ was standardised, calculated and recorded directly by the computerized ergospirometric system used (Aerograph 4.3, AeroSport Inc., Ann Arbor, MI, USA).

An Onyx 9500 fingertip pulse oximeter measured heart rate (HR; Nonin Medical Inc., Plymouth, MN, USA). The Borg scale measured rate of perceived exertion [12].

### Protocol

Anthropometric characteristics (height in m, weight in kg) were measured, and body mass index (BMI; kg·m^−2^) was calculated from them. Volunteers were first familiarised with the equipment, as well as both terrestrial CPR and ER techniques; volunteers were required to demonstrate that they had mastered both BLS methods.

Volunteers rested for 5 min prior to BLS to record baseline values. They then performed four sets of ECCs over a period of 1.5 min in accordance with the 2005 and 2010 ECC guidelines at 1G_z_ followed by the two gravitational simulations. A minimum of 10 min rest was given to volunteers between each set of ECCs.

ECC frequency and depth, as well as angle of elbow flexion, were measured throughout the experiment. Exhaled gases were sampled continuously and analysed every three breaths. Heart rate was recorded before (resting heart rate) and immediately after the completion of each protocol. After four sets of ECCs, subjective appraisal of exertion using the Borg scale was noted.

The Aerosport VO2000 analyser used its own software and was auto-calibrated prior to each protocol. The mannequin's chest system was calibrated between volunteers using inputs of 0 and 60 mm. The elbow electrogoniometer was calibrated prior to each protocol using two points: full extension of the arm (0°) and measured 90° flexion.

A DataQ acquisition device with eight analogue and six digital channels, 10 bits of measurement accuracy, rates up to 14,400 samples·s^−1^ and USB interface was used (DATA-Q Instruments Inc., Akron, OH, USA). The device supported a full-scale range of ±10 V and a resolution of ±19.5 mV. WinDaq data acquisition software allowed for the conversion of volts to the necessary units used. Two input channels were used during data collection: one from the chest system of the mannequin and the other from the elbow electrogoniometer.

### Data analysis

Data of physiological variables, which were determined by either averaging the last 30 s of exercise or comparing the last 30 s of exercise to baseline state and ECC depth, rate and elbow flexion, were reported as mean values (±SD). Percentage of maximum HR was calculated by comparing post-ECC HR with theoretical maximum HR (calculated using the 220-age equation) [13]. VO_2_peak represents the highest recorded VO_2_ during the four ECC sets. Elbow flexion was calculated as a range from the minimum to maximum angle of an individual ECC. The ECC depth was analysed in two different ways: maximum depth (*D*_Max_) achieved and true depth (*D*_T_), which was calculated using Equation 3:

(3) $$D_{T}=D_{\mathrm{Max}}-D_{\mathrm{IRecoil}}$$

where *D*_T_ is the true depth of external chest compression, *D*_Max_ is the maximum depth of external chest compression and *D*_IRecoil_ is the depth of inadequate recoil, which is the distance not decompressed between subsequent external compressions.

The measures were derived *post hoc* from the data files using GraphPad Prism v5.0a for analysis. Statistical comparisons were performed on physiological variables using a one-way, non-parametric ANOVA test and on ECCs and elbow flexion data using a two-way ANOVA. A 95% confidence interval calculation around the mean was used. The level of significance was set *a priori* as *p* ≤ 0.05.

## Results

All 30 volunteers completed the protocol. Mean (±SD) age, weight, height and BMI were 22.5 (±3.5) years, 78.2 (±13.1) kg, 1.80 (±0.07) m and 23.3 (±2.9) kg·m^−2^, respectively.

**Figure 2:**
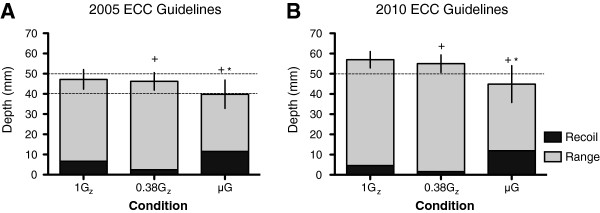
**Mean (±SD) maximum depth with depth of compressed chest post-inadequate recoil at 1G**_**z**_**, 0.38G**_**z **_**and μG.** (**A**) The 2005 ECC guidelines. (**B**) The 2010 ECC guidelines. The *dashed line(s)* depicts the effective limit(s) of depth for each respective guideline. *n* = 30; *asterisk* denotes significant difference in maximum depth to 1G_z_ control, *p* < 0.05. The *plus sign* denotes significant difference in recoil to 1G_z_ control, *p* < 0.05.

The mean (±SD) *D*_Max_ of all four sets for 1G_z_ and the simulated gravitational environments for the 2005 and 2010 ECC guidelines is presented in Figure 2A,B. All volunteers were able to abide by the 2005 and 2010 ECC guidelines at 1G_z_ (47.1 (±3.0) and 57.0 (±2.3) mm) and simulated 0.38G_z_ (46.2 (±3.6) and 55.1 (±3.7) mm). For simulated μG, the mean ECC *D*_Max_ obtained using the ER method fell 0.2 mm below the 2005 guidelines (39.8 (±8.3) mm), and there was considerable variation in the range of ECC *D*_Max_. Eleven volunteers were able to adhere to the 2010 ECC guidelines in simulated μG, and the mean *D*_Max_ fell short of the 50-mm effective limit (44.9 (±10.9) mm). However, not all volunteers allowed full recoil of the mannequin's chest for the three gravitational conditions. The mean (±SD) *D*_IRecoil_ for 1G_z_, 0.38G_z_ and μG were 6.7 (±4.9), 2.5 (±2.2) and 11.5 (±5.5) mm for 2005 ECC guidelines and 4.6 (±3.5), 1.6 (±1.8) and 11.9 (±5.7) mm for the 2010 ECC guidelines, respectively. For both ECC guidelines, *D*_IRecoil_ was less during the Martian simulation and higher during simulated μG.

**Figure 3:**
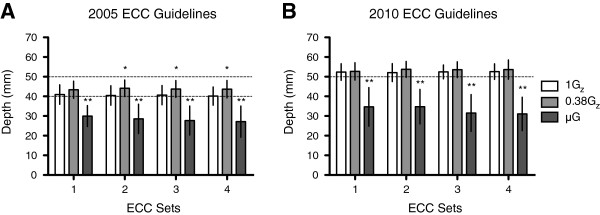
**Mean (±SD) true depth (*D***_**T**_**) of ECC at 1G**_**z**_**, 0.38G**_**z **_**and μG.** (**A**) The 2005 ECC guidelines. (**B**) The 2010 ECC guidelines. The *dashed line(s)* depicts the effective limit(s) of depth for each respective guideline. *n* = 30; *single asterisk* denotes *p* < 0.05, while *double asterisk* denotes *p* < 0.001.

The mean (±SD) *D*_T_ of the individual ECC sets per condition, calculated from *D*_IRecoil_ to *D*_Max_, is depicted in Figure 3A,B and Table 1. Mean *D*_T_ was within the effective limits set by the 2005 and 2010 ECC guidelines in the 1G_z_ control environment for all four ECC sets. For the 2005 ECC guidelines, the mean *D*_T_ for the last three ECC sets was above the effective lower limit for simulated 0.38G_z_ compared to 1G_z_. In contrast, the mean *D*_T_ of ECCs was below the effective lower limit in simulated μG compared to the 1G_z_ control environment for both ECC guidelines and for all four ECC sets.

**Table 1:** **Mean (±SD) true depth (*D***_**T**_**) and rate of individual ECC sets at 1G**_**z**_**, 0.38G**_**z **_**and μG**

**Gravitational condition**	**ECC guidelines**	**ECC**	**ECC sets**			
			**1**	**2**	**3**	**4**
1G_z_	2005	Depth (mm)	40.9 (±5.0)	40.4 (±5.0)	40.6 (±4.9)	40.1 (±4.6)
		Rate (comp·min^−1^)	104 (±5)	105 (±5)	105 (±6)	105 (±5)
	2010	Depth (mm)	52.4 (±4.2)	52.1 (±4.6)	52.5 (±3.5)	52.6 (±3.9)
		Rate (comp·min^−1^)	105 (±4)	104 (±4)	104 (±3)	104 (±3)
0.38G_z_	2005	Depth (mm)	43.4 (±4.4)	44.1 (±4.2)*	43.7 (±4.3)*	43.7 (±4.4)*
		Rate (comp·min^−1^)	103 (±6)	104 (±6)	104 (±5)	103 (±5)
	2010	Depth (mm)	52.7 (±4.4)	53.7 (±4.0)	53.6 (±4.0)	53.6 (±4.9)
		Rate (comp·min^−1^)	103 (±6)	103 (±5)	103 (±5)	103 (±5)
μG	2005	Depth (mm)	30.0 (±5.3)**	28.5 (±7.5)**	27.7 (±7.4)**	27.1 (±7.9)**
		Rate (comp·min^−1^)	105 (±7)	106 (±5)	105 (±5)	106 (±5)
	2010	Depth (mm)	34.7 (±9.8)**	34.8 (±8.7)**	31.5 (±9.4)**	31.1 (±8.5)**
		Rate (comp·min^−1^)	104 (±7)	105 (±5)	106 (±8)	103 (±10)

*n* = 30; *p < 0.05 and **p < 0.01, significant difference from 1G_z_ control.

The mean (±SD) ECC rate was successfully maintained above 100 compressions·min^−1^ for each set within each gravitational condition, with reference to both ECC guidelines (Table 1).

**Figure 4:**
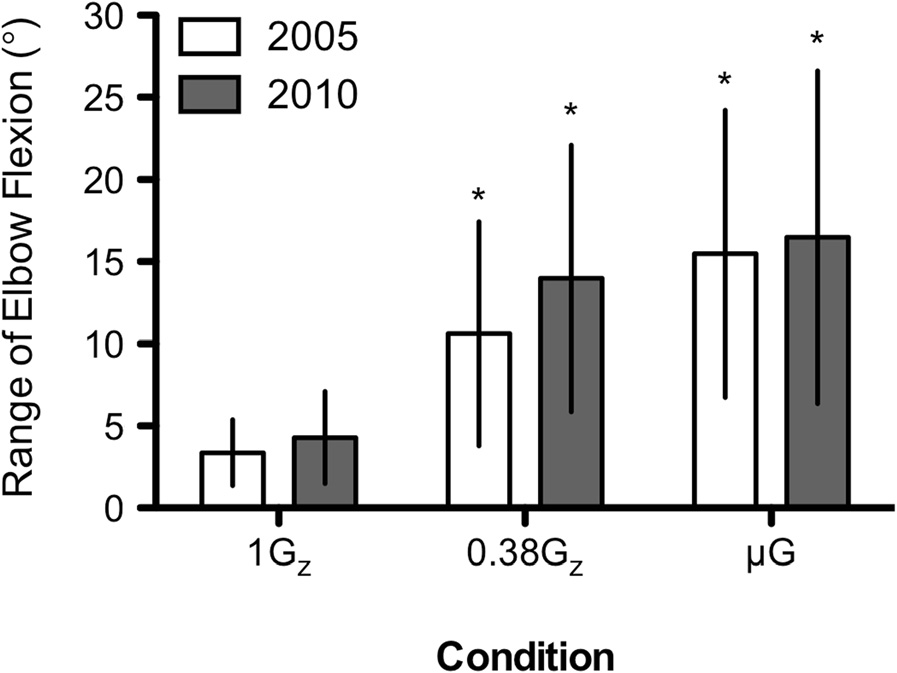
**Mean (±SD) range of elbow flexion in dominant arm at 1G**_**z**_**, 0.38G**_**z **_**and μG.***n* = 30; *Asterisk* denotes significant difference to 1G_z_ control, *p* < 0.05.

The mean (±SD) ranges of elbow flexion of the volunteer's dominant arm at 1G_z_ during the 2005 and 2010 ECC guidelines were 3.4° (±2.0°) and 4.3° (±2.8°), respectively. The range of elbow flexion increased to 10.6° (±6.8°) during the 2005 ECC guidelines and to 14.0° (±8.1°) during the 2010 ECC guidelines in simulated 0.38G_z_. When using the ER method in simulated μG, ranges of elbow flexion of the volunteer's dominant arm during the 2005 and 2010 ECC guidelines were 15.5° (±8.7)° and 16.5° (±10.1)°. No difference in range of elbow flexion was observed between ECC guidelines for either simulated reduced gravity conditions (Figure 4).

The mean (±SD) rescuer HR at baseline, post-ECC, as well as percent change and percentage of maximum HR in all three gravitational conditions is illustrated in Table 2. There was an increment in HR responses post-ECC for simulated 0.38G_z_ and μG. No differences between ECC guidelines were noted.

**Table 2:** **Mean (±SD) heart rate responses at 1G**_**z**_**, 0.38G**_**z **_**and μG**

**Mean (±SD), bpm**					**ECC guidelines**
**Baseline**	**Heart rate**	**1G**_**z**_	**0.38G**_**z**_	**μG**	
84 (±15)	HR post-ECC	111 (±19)	132 (±23)*	159 (±19)*	2005
	%Δ	33.8 (±18.4)	60.0 (±25.8)*	94.7 (±33.9)*	
	%Max	56.1 (±9.4)	66.9 (±11.6)*	80.7 (±9.9)*	
	HR post-ECC	117 (±21)	140 (±21)*	163 (±18)*	2010
	%Δ	41.4 (±22.8)	71.2 (±30.5)*	98.8 (±35.4)*	
	%Max	59.2 (±10.9)	71.1 (±10.7)*	82.3 (±9.4)*	

HR responses are depicted as baseline and post-ECC values (bpm), percent change from baseline and percentage of maximum heart rate (maximum heart rate was calculated using the 220-age equation). *n* = 30; *p < 0.05, significant difference from 1G_z_ control.

**Figure 5:**
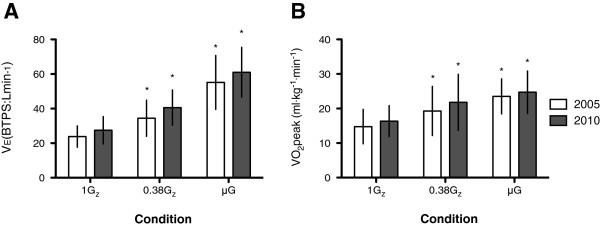
**Minute ventilation (*V***_**E**_**) and peak oxygen consumption (VO**_**2**_**peak) at 1G**_**z**_**, 0.38G**_**z **_**and μG.** (**A**) Mean (±SD) *V*_E_ at 1G_z_, 0.38G_z_ and μG. Baseline was 11.4 (±5.9) L·min^−1^. (**B**) Mean (±SD) VO_2_peak normalised to weight at 1G_z_, 0.38G_z_ and μG. Baseline was 3.2 (±1.1) mL·kg^−1^·min^−1^. *n* = 30; *Asterisk* denotes significant difference to 1G_z_ control, *p* < 0.05.

Mean (±SD) rescuer *V*_E_ for 1G_z_, 0.38G_z_ and μG increased from 11.4 (±5.9) L·min^−1^ at rest to 23.8 (±6.2), 34.4 (±10.4) and 55.1 (±15.6) L·min^−1^ for the 2005 ECC guidelines and 27.5 (±7.9), 40.6 (±10.2) and 61.1 (±14.4) L·min^−1^ for the 2010 ECC guidelines, respectively. With respect to both ECC guidelines, there was no significant difference in the increase in *V*_E_ from rest for the three gravitational conditions (Figure 5A). During the last 30 s of ECCs, *V*_E_ increased by 153.0%, 275.8% and 490.1% at 1G_z_, 0.38G_z_ and μG for the 2005 ECC guidelines. An increase of 194.8%, 334.9% and 568.1% was seen for the 2010 ECC guidelines, respectively.

During the performance of ECCs, the mean (±SD) rescuer VO_2_ increased from 3.2 (±1.1) mL·kg^−1^·min^−1^ at rest to peak levels of 14.8 (±5.0) mL·kg^−1^·min^−1^ at 1G_z_, 19.3 (±7.1) mL·kg^−1^·min^−1^ at 0.38G_z_ and 23.5 (±5.1) mL·kg^−1^·min^−1^ at μG for the 2005 ECC guidelines. For the 2010 ECC guidelines, the increase was to 16.4 (±4.5) mL·kg^−1^·min^−1^ at 1G_z_, 21.8 (±8.1) mL·kg^−1^·min^−1^ at 0.38G_z_ and 24.7 (±6.2) mL·kg^−1^·min^−1^ at μG (Figure 5B). During the last 30 s of ECCs, VO_2_ increased by 283.3%, 428.6% and 559.7% at 1G_z_, 0.38G_z_ and μG for the 2005 ECC guidelines. An increase of 367.7%, 509.0% and 590.3% was seen for the 2010 ECC guidelines, respectively. No difference was noted between ECC guidelines for all three gravitational conditions.

**Figure 6:**
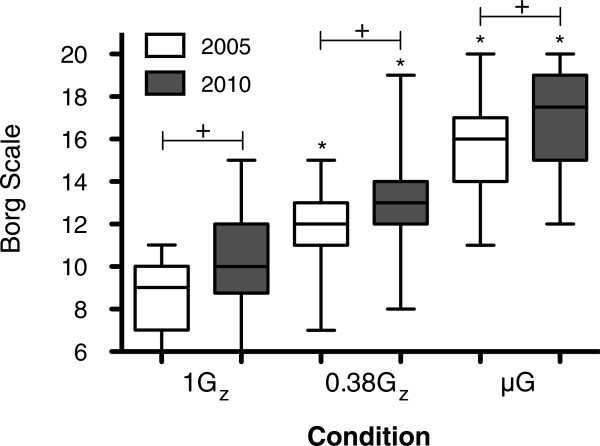
**Mean (±SD) rate of perceived exertion for four sets of ECCs at 1G**_**z**_**, 0.38G**_**z **_**and μG.***n* = 30; *Asterisk* denotes significant difference to 1G_z_ control, *p*<0.05. *Plus sign* denotes significant difference between ECC guidelines, *p* < 0.05.

The Borg scale showed there was an increase in the mean (±SD) rate of perceived exertion intra- and inter-conditions (Figure 6).

## Discussion

Preparation for adverse cardiac events is vital to ensure the safety of space explorers, thus potentiating the development of the most effective protocol for BLS in simulated 0.38G_z_ and μG.

This study was the first of its kind to investigate the administration of effective ECCs using the 2010 ECC guidelines in comparison to the previous 2005 ECC guidelines during simulated 0.38G_z_ and μG, while looking at the physiological impact on the rescuer.

Both ECC guidelines emphasise that effective ECCs have two key components—adequate compression depth and rate—to ensure sufficient haemodynamics from time of arrest to application of ALS. When assessing the *D*_Max_ achieved during ECCs, results from the 1G_z_ and simulated 0.38G_z_ sessions showed that all volunteers were able to perform according to both the 2005 and 2010 ECC standards. In fact, the ability of volunteers to abide by the previous 2005 ECC guidelines at 1G_z_ and during simulated 0.38G_z_ is in agreement with previous studies [10,14]. Although mean *D*_Max_ did not meet the 2005 ECC guidelines during simulated μG, a negligible difference of 0.2 mm would probably be deemed effective during *in vivo* BLS. However, the considerable inter-volunteer variability observed questions the efficacy of the ER method, which concurred with the findings of Rehnberg et al. [14]. Mean *D*_Max_ failed to abide by the 2010 ECC guidelines, which corresponds with the findings of Kordi et al. [15] (Figure 2B).

Previous studies noted that volunteers were failing to consistently allow full chest recoil using the ER method during simulated μG (Figure 2) [14]. This can be detrimental to the effectiveness of BLS, as incomplete decompression decreases the change in thoracic pressure and thereby reduces perfusion to vital organs. To address this issue and a first for space CPR studies, ECC *D*_T_ was calculated by adjusting for *D*_IRecoil_ (Figure 3 and Table 1).

When assessing ECC *D*_T_, all 30 volunteers failed to abide by both ECC guidelines during simulated μG using the ER method. This could be attributable to rescuers inadequately decompressing between individual ECCs or interruptions during ECCs when using the ER position in simulated μG.

The inadequate decompressions between ECCs may be due to rescuers focussing on achieving the 100 compressions·min^−1^ rate set by guidelines during simulated μG. This is supported by mean ECC rate in keeping with both sets of ECC guidelines, whilst true depth of individual ECC sets was not (Table 1). This is not in accordance with a previous parabolic flight study using the ER method that found ECC rate to be lower whilst ECC depth remained adequate for the used ECC guidelines at that time, which were the same as 2005. These findings, however, may represent a limitation of the BSD system. The parabolic study had a sample size of 3 and was able to adhere to ECC guidelines even with such a small window of freefall, approximately 20 s per parabola [11].

In addition, the high SD seen in Table 1, which represents the inter-volunteer variability for ECC rate, increases with time in simulated μG. This suggests degradation of ECC rate during the course of BLS.

Overall, the results of this investigation suggest that the ECCs administered were ineffective in simulated μG, as only mean ECC rate was adhered to, which would reduce the benefit to the casualty due to inadequate vital organ perfusion. This also indicates that the efficacy of the ER method is deficient at producing true depth of ECCs in a simulated μG environment, which contradicts previous studies only analysing maximum depth of ECCs and that did not account for *D*_IRecoil_ of the mannequin [14].

The efficacy of ECCs is dependent on the physiological impact of CPR on the rescuer. Increased HR, *V*_E_ and VO_2_peak were inversely correlated with the simulated gravitational conditions studied, which indicates greater physical effort during the performance of BLS (Table 2 and Figure 5). The HR results support those found by both Dalmarco et al. [10] and Rehnberg et al. [14]. Furthermore, there was no difference in HR, *V*_E_ and VO_2_peak between ECC guidelines, which may imply that current ECC guidelines do not impose additional physical effort, unlike the simulated gravitational environment.

In our study, it is important to note that VO_2_peak was measured and used as an estimation of VO_2_max, allowing comparisons with previous literature findings to be drawn [16].

There are limited studies that evaluate VO_2_max during or post-spaceflight, all of which are short-duration missions (<14 days) [17]. It has been hypothesised that appropriate exercise countermeasures may maintain VO_2_max during long-duration explorer-class missions. Thus, the additive effects of cardiac deconditioning would have less influence on the rescuer's aerobic capabilities to perform ECCs in μG, making the physical difficulty of the ER method the key variable in performing effective ECCs.

Interestingly, decreases in VO_2_max arise following re-entry. Levine et al. [18] noted that after the SLS-1 and SLS-2 missions, six astronauts showed VO_2_max levels of 2.1–2.9 L·min^−1^. In addition, the extra-vehicular activity (EVA) suit required for planetary surface exploration may also determine the level of cardiovascular exercise capacity. Studies at NASA's Johnson Space Center in simulated 0.38G_z_ showed an increase in VO_2_ by an additional 20 mL·kg^−1^·min^−1^ (40% of the volunteer's VO_2_max) while wearing a Mark III prototype exploration EVA suit [19].

After a Martian landing, crewmembers will most likely be required to begin work immediately without a sufficient period for acclimatisation to 0.38G_z_[20]. This reduced aerobic capacity, in conjunction with orthostatic intolerance and impaired blood flow from long-term microgravity exposure, may significantly impact a crewmember's capability during emergencies or while assisting an incapacitated crewmate.

Although any attempt to administer CPR in an EVA suit is unlikely to be achievable, the physiological aspect would be interesting to consider. Therefore, the mean VO_2_peak of a rescuer in an EVA suit would be 41.8 mL·kg^−1^·min^−1^ in simulated 0.38G_z_, which accounts for our 21.8 mL·kg^−1^·min^−1^ (Figure 5B) and the expected additional 20 mL·kg^−1^·min^−1^ from wearing an EVA suit [19]. For the average male weight (78.2 kg) in our study, this would equate to 3.3 L·min^−1^ after four sets of ECCs. This exceeds the VO_2_max of 2.9 L·min^−1^ found by Levine et al. [18]. This value may be an underestimation, as the casualty would also be wearing an EVA suit and the pressurisation of their suit would have to be overcome as well.

Furthermore, this study looked at the range of elbow flexion, while previous studies only took maximum elbow flexion into account [10,14]. The greater range of elbow flexion seen during simulated 0.38G_z_ could be accredited to the recruitment of upper arm muscle groups to compensate for the reduction in upper body weight. The lack of difference in the range of elbow flexion for either ECC guidelines may indicate that the upper limb muscle groups are recruited in the same manner (Figure 4).

An increase in elbow flexion range was also noted between simulated μG and 1G_z_. Using the 2005 ECC guidelines during simulated μG, the mean increase of 15.5° (±8.7°) in the volunteer's dominant arm was similar to the approximate 11° (±8.3°) and 15° (±9.1°) of the right and left arms, respectively, found by Rehnberg et al. [14]. However, it is important to highlight that the change from the terrestrial to the ER BLS position might have contributed to the recruitment of different muscle groups. Like simulated 0.38G_z_, the lack of difference in the range of elbow flexion using the ER method for both ECC guidelines may indicate that the upper limb muscle groups are recruited in the same manner. This further suggests that guidelines are equally difficult in simulated μG, as this correlates with the inability to achieve effective true ECC depth and the non-significant difference in physiological variables between guidelines (Figure 4).

Although the physiological variables measured were not different between guidelines, volunteers perceived current ECC guidelines to be more difficult (Figure 6). This might have been influenced by the fact that volunteers had a pre-conception that illuminating more LEDs for current ECC guidelines could have been less attainable.

This study is not without limitations, since it is based on the evaluation of healthy young males performing 1.5 min of BLS. The simulated gravitational environment, using a BSD, may not replicate all physiological effects secondary to reduced gravity exposure, apart from weight reduction, which is essential for successful BLS. This also applies to the mannequin when considering that chest wall expansion would occur upon reduced gravity exposure, affecting chest compression depth. Other psychological and physiological factors may differ in a simulated study compared to an actual cardiac arrest, such as stress. Furthermore, there are differences in chest wall compliance between humans and mannequins, which do not take into account variations in body anthropometrics, as well as EVA suits. In addition, the sample may not be representative of the commercial space passenger population in terms of demographics.

## Conclusion

In summary, the physiological variables measured showed no significant difference between the 2005 and 2010 BLS guidelines for all three gravitational conditions studied, although the performance of ECCs during hypogravity and microgravity simulations depicted an increase in physiological cost compared to terrestrial BLS.

This investigation demonstrated that despite ECC *D*_T_ and rate being in accordance to the 2005 and 2010 guidelines, accomplishing ECCs in a Martian environment might require a supra-maximal aerobic capacity. Further research into BLS and EVA suits is required to facilitate it on Mars.

Our study also showed that effective ECCs were not attainable for both the ECC guidelines in simulated μG using the ER BLS method. This indicates that future implementation of BLS education using the ER method in simulated μG and upper arm strength training are required to perform effective BLS in space.

Space agencies, commercial space ventures and academic institutions need to collaborate to devise a suitable BLS protocol for hypogravity and microgravity environments, accounting for the difficulty in meeting current terrestrial ECC guidelines in simulated reduced gravity conditions. These findings are even more pertinent with the dawn of commercial spaceflight.

## Abbreviations

ALS: Advanced life support; A-B-C: Airways-breathing-circulation; BLS: Basic life support; BMI: Body mass index; BSD: Body suspension device; CPR: Cardiopulmonary resuscitation; DIRecoil: Depth of inadequate recoil; ER: Evetts-Russomano; ECC: External chest compression; EVA: Extravehicular activity; HR: Heart rate; 0.38Gz: Martian hypogravity; DMax: Maximum depth; μG: Microgravity; VE: Minute ventilation; VO2 peak: Peak oxygen consumption; PUCRS: Pontifícia Universidade Catolica do Rio Grande do Sul; 1Gz: Terrestrial; DT: True depth.

## Competing interests

The authors declare that they have no competing interests.

## Authors’ contributions

TR participated in the design and coordination of the study. JHB participated in the design and coordination of the study, data collection and analysis and helped draft the manuscript. RV participated in the design and coordination of the study, data collection and helped draft the manuscript. RCB participated in data analysis of external chest compressions. AA participated in the data collection and analysis and helped draft the manuscript. LR participated in the data analysis of external chest compressions. RDG recruited volunteers and participated in the data collection and analysis. MKPD recruited volunteers and participated in the data collection and analysis. RRB participated in the design and coordination of the study. All authors read and approved the final manuscript.
